# Inflammasome and Cognitive Symptoms in Human Diseases: Biological Evidence from Experimental Research

**DOI:** 10.3390/ijms21031103

**Published:** 2020-02-07

**Authors:** So Yeong Cheon, Jeongmin Kim, So Yeon Kim, Eun Jung Kim, Bon-Nyeo Koo

**Affiliations:** 1 Anesthesia and Pain Research Institute, Yonsei University College of Medicine, Seoul 03722, Korea; 2 Department of Anesthesiology and Pain Medicine, Yonsei University College of Medicine, Seoul 03722, Korea

**Keywords:** inflammasome, cognitive symptoms, neurodegenerative disease, inflammatory disease, IL-18, IL-1β, caspase-1

## Abstract

Cognitive symptoms are prevalent in the elderly and are associated with an elevated risk of developing dementia. Disease-driven changes can cause cognitive disabilities in memory, attention, and language. The inflammasome is an innate immune intracellular complex that has a critical role in the host defense system, in that it senses infectious pathogen-associated and endogenous danger-associated molecular patterns. An unbalanced or dysregulated inflammasome is associated with infectious, inflammatory, and neurodegenerative diseases. Due to its importance in such pathological conditions, the inflammasome is an emerging drug target for human diseases. A growing number of studies have revealed links between cognitive symptoms and the inflammasome. Several studies have shown that reducing the inflammasome component mitigates cognitive symptoms in diseased states. Therefore, understanding the inflammasome regulatory mechanisms may be required for the prevention and treatment of cognitive symptoms. The purpose of this review is to discuss the current understanding of the inflammasome and its relationships with cognitive symptoms in various human diseases.

## 1. Introduction

Cognitive impairment is common, and its prevalence is growing with the increase in the elderly population [1]. In particular, pathological changes during disease processes are closely related to the loss of cognitive abilities (memory, attention, and language) [2]. Many patients suffer from cognitive impairment, which can result in the development of dementia [1,2]. The incidence of dementia continues to increase from the age of 65 to 90 years and doubles every 5 years [3,4]. Pathologic phenotypes such as amyloid plaque, neurofibrillary tangles, Lewy bodies, and macro- and micro-infarctions are observed in the brain of elderly individuals [1,5,6]. Moreover, early activation of the microglia and peripheral blood inflammatory changes are involved in cognitive decline and dementia [7,8].

Innate immunity is the first responder in the host defense system and monitors and clears both sterile and infectious signs [9]. Innate immune cells expressing pattern recognition receptors (PRRs) (nucleotide-binding oligomerization domain (NOD)-like receptors (NLRs), retinoic acid-induced gene (RIG)-I-like receptors (RLRs), absent in melanoma 2 (AIM2)-like receptors (ALRs), Toll-like receptors (TLRs), and C-type lectin receptors (CLRs)) can sense infectious foreign pathogen-associated molecular patterns (PAMPs) and damaged or dying cell-derived endogenous danger-associated molecular pattern (DAMPs) [10,11]. Among them, the cytosolic NLR and ALR families form an intracellular complex—the inflammasome—which binds through an adaptor protein, the apoptosis-associated speck-like protein containing a carboxy-terminal CARD (ASC) and subsequently caspase-1 [9,12]. The inflammasome plays pivotal roles in the maturation of pro-inflammatory interleukin (IL)-1β and IL-18 and triggers pyroptosis, an inflammatory form of cell death [13].

The inflammasome is implicated in anti-microbial defense and tissue repair [14]. However, it can drive human diseases, including inflammatory and neurodegenerative diseases. The association of the inflammasome with cognitive symptoms and inflammasome-associated pathophysiology have been reported in several pathological conditions [15,16,17,18,19]. Therefore, understanding the mechanisms regulating the inflammasome complex may be important for preventing or treating cognitive disorders. This review aims to discuss the current understanding of the inflammasome and its relationships with cognitive symptoms in various human diseases.

## 2. Brief Overview of the Inflammasome

Cytosolic scaffold proteins of the NLR or ALR families, such as NLRP1, NLRP2, NLPR3, NLRC4, NLRP6, NLRP7, NLRP12, and AIM2, are involved in inflammasome assembly [20,21]. A protein of the NLR or ALR family can bind to the N-terminal pyrin domain (PYD) of ASC. The C-terminal caspase activation and recruitment domain (CARD) of ASC can activate caspase-1 by linking pro-caspase-1 into the complex [9,12]. Caspase-1 regulates the maturation of pro-inflammatory IL-1β and IL-18 [13]. Canonical inflammasome-mediated caspase-1 and non-canonical inflammasome-mediated caspase-11 (human caspase-4 and 5) induce an inflammatory form of cell death, named pyroptosis [13,22,23]. Gasdermin D (GSDMD), cleaved by caspase-1 and caspase-11, is required during this process as an executor/executioner of pyroptosis [24,25].

**Figure 1:**
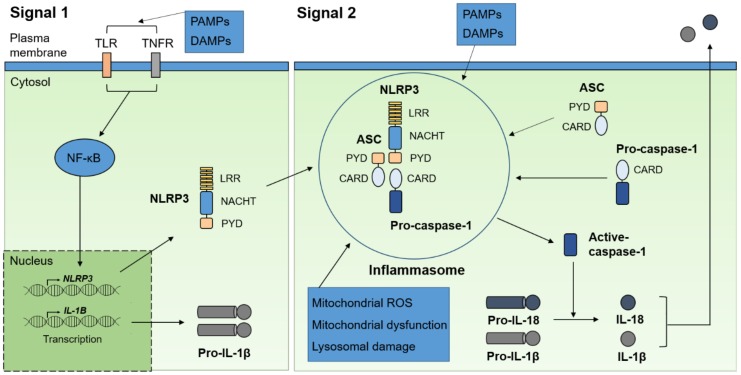
A two-step process for NLRP3 inflammasome activation. A priming signal is mediated by TLRs or TNFR, which can activate the NF-κB pathway. The NF-κB transcription factor upregulates the mRNA and protein levels of NLRP3 and pro-IL-1β. A second signal (Signal 2) is activated by various stimuli including PAMPs and DAMPs, and directly promotes NLRP3 activation and inflammasome assembly. Subsequently, the NLRP3 inflammasome is involved in the cleavage of pro-IL-1β and pro-IL-18, leading to their activation. PAMPs, pathogen-associated molecular patterns; DAMPs, danger-associated molecular patterns; TLRs; Toll-like receptors; tumor necrosis factor receptor (TNFR); NF-κB, nuclear factor kappa-light-chain-enhancer of activated B cells; LRR, leucine-rich repeat domain; NACHT (also known as NBD), nucleotide-binding domain; PYD, pyrin domain; CARD, caspase recruitment and activation domain; ROS, reactive oxygen species.

The NLRP3 inflammasome is formed by the NLRP3 protein, interacting through its PYD with ASC, which, in turn, binds pro-caspase-1 through its CARD. NLRP3 activation is triggered by various stimuli, including PAMPs, DAMPs, ion fluxes, and aggregated substances [26,27]. A two-step process is required for NLRP3 activation: 1. A priming signal (mediated by TLR or TNFR) is involved in the upregulation of NLRP3 and pro-IL-1β levels through the activation of transcription factor nuclear factor kappa-light-chain-enhancer of activated B cells (NF-κB) and post-translational modifications of NLRP3. 2. A second signal induces and activates NLRP3 inflammasome assembly. NLRP3 inflammasome activation is closely associated with mitochondrial reactive oxygen species (ROS) production, mitochondrial dysfunction, and lysosomal damage (Figure 1) [26,27,28].

The human NLRP1 protein has both PYD and CARD domains. The mouse genome encodes three NLRP1 homologs, namely, NLRP1a, NLRP1b, and NLRP1c, which lack the PYD. They can bind pro-caspase-1 directly via their CARD, without interacting with ASC. Therefore, the NLRP1 inflammasome can lead to caspase-1 activation through an ASC-dependent manner or through direct CARD-CARD interaction. The *Bacillus anthracis* lethal factor can activate the NLRP1 inflammasome [9,27,29,30]. The involvement of NLRP1 in autoimmune diseases has been suggested [30,31,32,33].

The NLRC4 protein has a CARD, which directly interacts with the CARD of pro-caspase-1, without ASC, to form the inflammasome. Likewise, ASC can enhance the secretion of IL-1β and IL-18 in the NLRC4 inflammasome [34]. A family of NLR apoptosis inhibitory proteins (NAIPs) can sense flagellin and type-III secretion system (T3SS) components in the cytosol during infection by bacteria such as *Salmonella typhimurium* and *Shigella flexneri*. The NAIPs can recruit NLRC4 to form the inflammasome assembly [26,34,35]. The NLRC4 inflammasome is involved in the recognition of intracellular bacterial pathogens and regulation of enteric infections [35,36]. NLRC4 has also been associated with autoinflammatory diseases [35,37].

**Figure 2:**
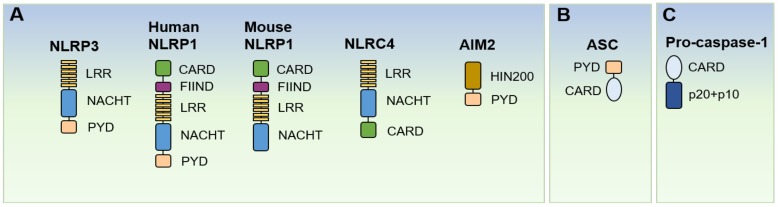
Schematic structure of NLRPs, ASC, and caspase-1. (**A**) Structures of inflammasome sensors NLRP3, NLRP1, NLRC4, and AIM2. (**B**) Structures of inflammasome adaptor protein ASC and (**C**) inflammasome effector pro-caspase-1. LRR; leucine-rich repeat domain, NACHT (or NBD); nucleotide-binding domain, PYD; pyrin domain, CARD; caspase recruitment domain family, FIIND; function-to-find domain, HIN200; 200-amino acid repeat.

Moreover, AIM2 consists of a PYD and a DNA-binding HIN200 domain, so that it can recognize cytosolic double-stranded DNA via the HIN200 and recruit ASC via the PYD. Microbial DNA from intracellular pathogens (vaccinia virus (VACV), *Francisella tularensis*, *Mycobacterium*, and *Listeria monocytogenes*), type I interferons, and DNA from the host itself are critical for the activation of AIM2 inflammasome [38,39,40,41], which is essential for gut microbiota and intestinal homeostasis. Human inflammatory and autoimmune diseases such as psoriasis, systemic lupus erythematosus, and atopic dermatitis are related to AIM2 inflammasome (Figure 2) [41,42].

NLRP6 can recognize enteric viruses and interact with viral RNA via RNA helicase Dhx15, an RNA sensor, to promote type I/III interferons (IFNs) and IFN-stimulated genes [43]. NLRP6 is abundantly detected in the intestinal epithelium and is important for colonic microbiota and gut immunity. NLRP6 in the intestinal epithelium is involved in the regulation of autophagy [44].

## 3. Sepsis-Associated Encephalopathy

Sepsis is a condition caused by an infection (bacterial, fungal, or viral), leading to organ dysfunction [45,46,47], and sepsis-associated encephalopathy (SAE) is a common complication of sepsis [46,48]. SAE occurs in a large proportion (up to 70%) of patients in an intensive care unit suffering from severe systemic inflammation [48]. Severe SAE patients have higher mortality rates [45,48]. SAE is characterized by brain impairment and has been suggested as a cause of altered neurological and mental states, including visuo-spatial deficits, memory loss, inattention, agitation, and disorientation. When severe, it can lead to stupor and coma [46,49,50,51]. Magnetic resonance imaging (MRI) and computerized tomography (CT) have shown that SAE manifests as extracranial abnormalities and neurological disease-like changes [52]. In addition, MRI has revealed diverse brain injuries, ischemic lesion, and white matter hyperintensities in the paraventricular or paramedian regions [53]. Along with morphological changes in the brain, cognitive dysfunctions, including substantial new cognitive deficits, can last long after hospitalization and eventually develop into dementia [54,55].

The pathophysiology of SAE has not been clearly elucidated yet, but an in vivo study of a cecal ligation and puncture (CLP) model mimicking human sepsis or SAE has demonstrated through MRI the presence of vasogenic and cytotoxic edema, as well as neuronal damage and brain injury [56]. Similarly, in another study, MRI revealed cytotoxic edema and brain injury in a CLP model. Activated microglia and inflammatory response were observed in several brain regions, such as the hippocampus, cortex, and thalamus, of CLP mice [57]. Lipopolysaccharide (LPS)-injected mice showed inflammation-induced blood-brain barrier (BBB) breakdown in specific brain regions, including the frontal cortex, striatum, thalamus, and hippocampus. Changes in the water content in this model may be due to the breakdown of the BBB. At the same time, inflammatory cytokines such as IL-lα, IL-1β, IL-3, IL-4, IL-9, IL-10, monocyte chemoattractant protein-1 (MCP-1), and granulocyte-macrophage colony-stimulating factor (GM-CSF) are abundantly expressed in the brain. In addition, inflammatory stimuli induce reactive microglia and astrocytes, as well as the activation of peripheral macrophages/monocytes [58]. Similarly, in a LPS injection model, the mice displayed breakdown of the BBB by detachment of pericytes from the basal lamina and reactive microglia in the brain lesion [59]. Mice treated with LPS show anxiety-like symptoms (as revealed by the elevated plus maze test) as well as depressive-like ones (tail suspension test and sucrose preference test). In these mice, microglial markers, such as CD11b, Iba-1, and F4/80, are highly expressed in the hippocampus; however, plasticity-related molecules and proliferating neural stem cells are decreased in the brain [60]. Reactive microglia, neuroinflammation, and apoptotic neuronal cell death have been suggested as potential causative factors of SAE [53].

Interestingly, the CLP model shows impaired spatial learning and memory in the Morris water maze test and microglial activation, NLRP3 expression, and IL-1β cleavage in the hippocampus. In a BV2 microglia cell line, LPS-treated microglia showed increased levels of NLRP3, caspase-1, and IL-1β [61]. LPS-exposed mice display loss of recognition memory in a novel object recognition test and long-term depression-like behaviors. In these mice, the NLRP3 complex components, including NLRP3, ASC, and caspase-1 p10, and levels of IL-18, IL-1β, and tumor necrosis factor-α (TNF-α) are increased in the hippocampus where microglial activation is observed. However, these symptoms are reduced by a caspase-1 specific inhibitor, which suppresses NLRP3 inflammasome activity [15]. Mice subjected to CLP exhibit hippocampal memory impairment in a fear conditioning test, damaged hippocampal structure, activation of the NLRP3/caspase-1 pathway, increased pyroptosis, and inflammatory cytokines such as IL-1β and IL-18; however, NLRP3 inhibition reversed these outcomes after CLP injury [62], suggesting the involvement of NLRP inflammasome in SAE-associated cognitive disorders.

In SAE models, resveratrol, Ac-Tyr-Val-Ala-Asp-chloromethylketone (Ac-YVAD-CMK), and MCC950 show protective effects against inflammasome and cognitive dysfunction [15,61,62]. Resveratrol is a natural phenol known as a potential therapeutic agent in neurodegenerative diseases. It is involved in the activation of sirtuin 1 (Sirt1) and inactivation of inflammatory proteins. In both in vivo and in vitro systems, resveratrol inhibits NLRP3, caspase-1, and IL-1β expression in microglia cell lines. It also suppresses the NLRP3 inflammasome and IL-1β in the hippocampus, thereby reducing spatial learning and memory in CLP mice [61]. Ac-YVAD-CMK, a caspase-1 inhibitor, can reduce the expressions of NLRP3 inflammasome, IL-1β, and IL-18 in the hippocampus and attenuate memory deficits and emotional abnormalities [15]. MCC950, an NLRP3 inhibitor, or Ac-YVAD-CMK ameliorates neuronal pyroptosis and neuroinflammation in the hippocampus, thereby decreasing cognitive impairment [62].

## 4. Perioperative Neurocognitive Disorders

Cognitive decline in patients following anesthesia and surgery is a common clinical symptom, which may be a risk factor for dementia and death [63]. Following surgical procedures, the incidence of cognitive impairment is estimated to be about 25% at 1 week and 10% at 3 months [64]. Among the types of cognitive impairment seen during the perioperative period, delirium (acute confusional state) is characterized by disturbed attention, cognition, and awareness [65]. Broadly defined, postoperative cognitive dysfunction (POCD) is any decrement in cognition, such as learning and memory, perception, and executive functions [66]. From 2018, the Nomenclature Consensus Working Group recommends the use of the integrated term ‘perioperative neurocognitive disorders’ (PNDs) for cognitive impairment or changes, including POCD and acute delirium, during the perioperative or postoperative phase [67].

The pathophysiology of PNDs has not been fully clarified; however, inflammatory response, age, and anesthesia duration may be associated with PNDs [68,69,70,71]. Pain, disruption of circadian rhythms, and systemic inflammation in response to surgery, infection, or injury are regarded as causative factors of delirium, a specific form of PND [72,73,74,75]. Surgery-induced tissue damage may trigger the peripheral immune response and release of inflammatory mediators. In particular, aged mice showed neuroinflammation (upregulation of IL-1β) in the hippocampus and reduced cognitive flexibility in the Morris water maze after minor abdominal surgery. However, anesthetics and analgesics are not correlated with neuroinflammation [68]. Aged mice subjected to abdominal surgery displayed increased hippocampal β-amyloid (Aβ) and beta-site APP cleaving enzyme (BACE1) levels in the hippocampus and cognitive impairment, while general anesthesia did not cause such cognitive deficits [76]. Abdominal surgery is involved in peripheral inflammation and infiltration of immune cells via the disruption of the BBB, leading to neuroinflammation and cognitive impairment [69]. Moreover, orthopedic surgery results in systemic inflammation, followed by IL-1β-mediated inflammation in the plasma and hippocampus, and deficits in contextual fear memory. Such inflammatory response and hippocampal microgliosis were reduced in IL-1 receptor (IL-1R) antagonist (IL-1RA)-treated mice or IL-1R deficient mice [70]. Mice subjected to tibial fracture surgery exhibited neuroinflammation and cognitive impairment due to TLR4/MyD88 signaling-mediated upregulation of S100A8 in the peripheral blood mononuclear cells (PBMCs), spleen, and hippocampus; however, microgliosis was reduced in both TLR4-deficient and MyD88-deficient mice and anxiety/cognitive dysfunction was reversed in TLR4-deficient mice [71].

Moreover, the ME7 mouse model of prion diseases with systemic inflammation induced by LPS exhibits acute and transient cognitive dysfunction, related to the pre-existing synapse loss and microglia priming in the hippocampus. These mice also show markedly increased inflammatory mediators in the central nervous system (CNS) [72]. Upregulated hippocampal and hypothalamic expressions of IL-1β, TNF-α, and CCL2 were detected in ME7 mice after systemic injection of TNF-α, together with acute loss of cognition and sickness behavior [73]. Additionally, pain after abdominal surgery resulted in memory deficits and neuroinflammation in the medial prefrontal cortex and hippocampus, while analgesics such as ropivacaine and morphine alleviated these symptoms [74].

The inflammasome has been reported to be related to the pathogenesis of PND in various murine models [16,69,77,78]. In a mouse model subjected to anesthesia (isoflurane), aged mice, but not young ones, showed activation of the NLRP3-caspase-1 pathway, poor performance in the Morris water maze, and increased levels of IL-1β and IL-18 [16]. Moreover, in mice subjected to major abdominal surgery, components of the NLRP1 inflammasome complex are expressed and IL-1β and IL-18 are highly produced in the spleen, whereas the inhibition of the NF-κB p65 transcript reduced these effects [77]. Surgical operation/anesthesia (sevoflurane) increases the activation of NLRP3 inflammasome, leading to cognitive impairment. Interestingly, it was suggested that increased mitochondrial ROS and malondialdehyde (MDA) production is associated with the NLRP3 inflammasome [78].

Ac-YVAD-CMK, NF-κB p65 inhibition, and Honokiol contribute to reducing the inflammasome and ameliorating cognitive impairment in PND models [16,77,78]. Ac-YVAD-CMK can attenuate anesthesia (isoflurane)-induced activation of NLRP3 inflammasome in aged brain, thus ameliorating cognitive deficits [16]. Also, the inhibition of NF-κB p65, which is a transcriptional regulator for inflammatory mediators, results in the reduction of NLRP1 inflammasome constituents and IL-18 and IL-1β production in the spleen and reduction of cell death in the spleen. It also reduces long-term potentiation (LTP) deficits and memory impairment [69,77]. Honokiol, a poly phenolic compound, has anti-inflammatory, anti-oxidant, and anti-tumor properties and has shown inhibitory effects on NLRP3 inflammasome and restorative effects on cognitive impairment [78].

## 5. Multiple Sclerosis

Multiple sclerosis (MS) is a chronic auto-inflammatory demyelinating disease, in which nerves are damaged by demyelination in the CNS [79]. MS patients show abnormalities in motor function, muscle weakness, and sensorimotor dysfunction [80]. Brain MRI imaging reveals atrophy of both the white and gray matter, with demyelination and axonal degeneration [81]. Approximately 40–60% of MS patients develop cognitive impairments, affecting memory, executive function, and processing speed [82,83]. Inflammation, demyelination, mitochondrial dysfunction, and axonal loss are the main pathophysiological hallmarks of MS [84,85].

Many reports have discussed the association of inflammation and MS. During MS progression, axonal lesions are correlated with inflammation and inflammatory infiltrates [86]. In agreement with clinical observations, the experimental autoimmune encephalomyelitis (EAE) animal model of MS shows alteration of brain volumes in the chronic phase of the disease. For example, EAE mice undergo regional volume loss in both white and gray matter, including the hippocampus, cerebral cortex, and cerebellum, accompanied by axonal loss and demyelination [87]. EAE mice display activated microglia-derived IL-1β-dependent alterations in γ-aminobutyric acid (GABA)-ergic activity in the hippocampus, suggesting that pronounced aberrant synaptic plasticity could result in cognitive dysfunction [88]. As expected, LTP and spatial memory are impaired in the late stages of EAE [89]. Similarly, EAE mice show persistent microglial activation and subsequent impairment of long-term synaptic plasticity in the hippocampus. Such LTP damage is mediated by nicotinamide adenine dinucleotide phosphate (NADPH) oxidase and results in hippocampal-dependent cognitive dysfunction. Conversely, the inhibition of NADPH oxidase can recover LTP and inhibition of microglial activation also reverses synaptic and cognitive deficits [90].

The central role of the inflammasome has been demonstrated in MS [91,92]. Inflammasome-associated genes, including NLRP3, IL-1β, IL-18, caspase-1, GSDMD, and ASC, are increased in the postmortem brains of MS patients, and GSDMD is observed in the myeloid cells and oligodendrocytes of MS lesions, indicating pyroptosis. Similar to MS patients, EAE mice show activation of inflammasome, pyroptosis, and axonal injury in the CNS. Caspase-1 inhibition in EAE mice can reverse inflammasome activation, pyroptosis in the myeloid cells and oligodendrocytes, and neurobehavioral deficits [91]. Systemic or splenic IL-1β and IL-8 are increased in EAE mice; however, NLRP3- or ASC-deficient mice fail to induce IL-1β and IL-8 production [92]. EAE mice show increased NLPR3 expression in the spinal cord, with increased infiltration of immune cells (CD4 T cell, CD8 T cell, macrophages, and dendritic cells) and upregulated IL-18 in the serum and spinal cord. Genetic NLRP3 deficiency delays the development and decreases the severity of the disease by suppressing the immune infiltrates into the spinal cord and reducing T helper (Th)1 and Th17 T cell response, thus preventing the disruption of the myelin sheath [17]. EAE mice, before demyelination, exhibit anxiety- and depression-like behaviors and cognitive impairment, which are dependent on the increased IL-1β and TNF-α levels in the hypothalamus [93]. Overexpression of IL-1β leads to MS-like pathologic phenotypes, including neurodegeneration, demyelination, and inflammation, together with cognitive impairments [94].

In an MS model, caspase-1 inhibitor VX-765 reduced inflammasome- and pyroptosis-associated proteins in the spinal cord, alleviated neuropathological features such as axonal injury, and prevented neurobehavioral deficits [91].

## 6. Alzheimer’s Disease

Alzheimer’s disease (AD) is a neurodegenerative disease characterized by the progressive impairment of memory and cognition, which can develop into dementia [95]. Early onset familial AD is related to the mutation of genes, including presenilin 1 (PSEN1), presenilin 2 (PSEN2), and amyloid precursor protein (APP) [95]. Apolipoprotein E(APOE) is responsible for both sporadic AD and late-onset familial AD [95,96]. Despite such distinction, the clinical and pathologic phenotypes are similar between familial and sporadic AD [95]. Increasing evidence suggests that impaired autophagy, extracelluar Aβ deposition, intracellular neurofibrillary tangles of tau, and imbalanced immune response are the main biological features of AD [97,98]. After cleaving by beta- or gamma-secretase, APP produces Aβ, the main component of the amyloid plaque [99]. While tau, a major microtubule-associated protein (MAP), plays an important role in the stabilization of microtubules, its hyperphosphorylation can generate neurofibrillary tangles [100]. Normally, aggregate-prone proteins, including Aβ, are removed by autophagy [97]. Under compromised autophagy, such as in AD, the Aβ aggregates are markedly increased in the postmortem brain [97,101].

Immune cell activation and inflammatory response are observed as common hallmarks of AD [102]. The APP/PS1 and APP/PS1dE9 transgenic mouse models of AD display activated microglia and expresse pro-inflammatory cytokines such as IL-1β, CXCL1, CCL3, and CCL4 [103]. Pro-inflammatory IL-18 increases APP expression and Aβ formation in the neuronal SH-SY5Y cell line [104]. In a rat primary microglia and cortical neuronal cell culture, secreted APP alpha (sAPPα) from neuron can activate microglia, which release IL-1β. Increased level of IL-1β is associated with elevated levels of α-synuclein and tau hyperphosphorylation in neuron [105]. Interestingly, microglial LC3-associated endocytosis has been suggested to contribute to protection against Aβ deposition and inflammatory response in an AD model. Aβ_1–42_ exposure results in enhanced expression of pro-inflammatory cytokines such as IL-1β, IL-6, and TNF-α in the BV2 microglia cell line and in primary microglia. Furthermore, deficiency in LC3-associated endocytosis-related genes, including Atg5 and Rubicon, exacerbates the levels of pro-inflammatory cytokines [106]. APPswe/PSEN1dE9 transgenic mice show loss of spatial learning and memory, increased anxiety, and defects in social interaction, which are associated with amyloid deposition in the cerebral cortex and hippocampus. In particular, reactive microglia and astrocytes are observed around these amyloid depositions, and the same cells are involved in Aβ-induced inflammatory response, producing IL-1β, IL-6, IL-17A, and TNF-α [107]. In addition, the APP21 transgenic rat model of AD, which expresses human APP, shows impaired executive functions, white matter microglial activation and inflammation, and vulnerability to amyloid generation [108].

According to some studies, NLRP3 inflammasome is present in AD [18,109]. In the brain of AD patients, high levels of cleaved (activated) caspase-1 are observed in the hippocampus and cortex. Accumulation of Aβ is involved in microglial activation, and Aβ-induced activation of the NLRP3 inflammasome is required for caspase-1 activation. The APP/PS1 mouse model of AD exhibits phagocytic dysfunction for Aβ, synaptic dysfunction, and spatial memory impairment; however, NLRP3 or caspase-1 deficiencies in these mice increase Aβ clearance, synaptic plasticity, and memory function [109]. The NLRP1 inflammasome has reported as highly expressed in response to Aβ accumulation in the brain of APPswe/PS1dE9 mice, and thus indicated as a mediator of Aβ neurotoxicity; indeed, APPswe/PS1dE9 mice show neuronal pyroptosis in the hippocampus and cortex and cognitive loss, while the genetic knockdown of NLRP1 or caspase-1 in this model reverses pyroptosis and cognitive dysfunction [110]. The microglia may also be critical to the formation of Aβ plaques by releasing inflammasome-dependent ASC specks, which directly bind to Aβ in APPswe/PSEN1dE9 mice. In contrast, genetic ASC deficiency in APPswe/PSEN1dE9 mice shows a beneficial role in the pathology of AD [111]. A previous study has reported that amyloid deposition and memory loss are increased in 5xFAD mice; however, ASC heterogeneity in these mice attenuates these outcomes [112]. Furthermore, the NLRP3 inflammasome is responsible for the pro-inflammatory and neurotoxic properties of Aβ via caspase-1 activation in the microglia stimulated with Aβ. A genetic deficiency in caspase-1 can ameliorate neurotoxic effects and chemokine levels even after exposure to Aβ, and the caspase-1 inhibitor z-YVAD-fmk attenuates IL-1β secretion in the microglia despite Aβ stimulation [18].

In addition, MCC950 and VX-765 have shown protective effects in AD models [113,114]. MCC950 improved disrupted synaptic plasticity and LTP in transgenic rat overexpressing APP [113]. The caspase-1 inhibitor VX-765 suppressed both neuroinflammation and accumulation of Aβ in a J20 APP ^Sw/Ind^ transgenic mouse model of AD. Also, VX-765 attenuated learning and memory impairment in this AD model [114].

## 7. Parkinson’s Disease

Parkinson’s disease (PD) is a progressive neurodegenerative disease featured by tremor, rigidity, cognitive decline, and behavioral difficulties [115]. PD is characterized by dopaminergic neuronal loss in the substantia nigra and intraneuronal Lewy bodies, mainly containing α-synuclein [115,116]. Furthermore, exosomal α-synuclein can confer cognitive impairment, even leading to dementia [117]. Both the ubiquitin-proteasome system (UPS) and autophagy may be involved in the degradation of α-synuclein. However, impairment of the protein degradation system can lead to the formation of neurotoxic α-synuclein aggregates [115,118,119]. It has been reported that oxidative stress and inflammation are one of the main causes of PD [120,121].

In particular, growing evidence shows distinct inflammatory reactions in the pathogenesis of PD [122]. Recruitment of peripheral immune cells, reactive microgliosis, and increased pro-inflammatory mediators are observed in the brain of PD patients and in animal model of PD [123,124,125]. In an intra-nigrally α-synuclein-injected model of PD, α-synuclein was shown to induce the infiltration of peripheral myeloid cells into the CNS, which have pro-inflammatory properties [123]. Extracellular α-synuclein is associated with the activation of microglia depending on NADPH oxidase activity and ROS production [126]. Similarly, α-synuclein transgenic mice show highly expressed reactive microglia in the substantia nigra and pro-inflammatory TNF-α. The microglia under synuclein-incubated conditions induces TNF-α, COX2, NOX2, and iNOS [127]. A rat model of PD using 6-hydroxydopamine (6-OHDA) presented impaired memory functions, increased reactive microglia, and imbalance between pro- and anti-inflammatory mediators [128]. A53T transgenic mice display motor and coordination impairment, consistent with the symptoms of PD patients. A53T mice also present memory impairment, long-term depression, and synaptic impairment [129]. A53T mice expressing mutant α-synuclein display increased inflammatory response, together with early cognitive impairment [130].

It has been proved that inflammasome-related signals such as NLRP3 and IL-1β are expressed in the serum, and NLRP3 inflammasome is closely correlated with α-synuclein in PD patients [131]. The previous study proved that in fibrillar α-synuclein or 6-OHDA injection mouse model of PD, the NLRP3 inflammasome acts as a bridge between fibrillar α-synuclein and dopaminergic neuronal loss. These mice show deficits in motor function and nigrostriatal dopaminergic neuron; however, the inhibition of NLRP3 protects them from these associated symptoms [19]. Interestingly, α-synuclein aggregation is influenced by caspase-1. A truncated form of α-synuclein, enriched in insoluble aggregates and found in Lewy bodies, is formed by active caspase-1. Genetic/pharmacological inhibition of caspase-1 plays an important role in reducing the truncation of α-synuclein in BE(2)-M17 neuronal cells overexpressing α-synuclein [132]. Also, α-synuclein is involved in IL-1β synthesis and secretion through inflammasome and caspase-1 activation [133]. In a 1-methyl-4-phenyl-1,2,3,6-tetrahydropyridine (MPTP)-induced PD model, mice showed severe motor deficit, loss of dopaminergic neurons, and increased α-synuclein; however, these phenotypes were reduced by inhibiting NLRP3 inflammasome activation [134].

In PD mouse models, MCC950 prevented the α-synuclein-induced activation of NLRP3 inflammasome, dopaminergic neuronal degeneration, and motor deficits [19]. Furthermore, VX-765 suppresses α-synuclein truncation and subsequent neurotoxicity by inhibiting caspase-1 [132].

## 8. Conclusions

**Figure 3:**
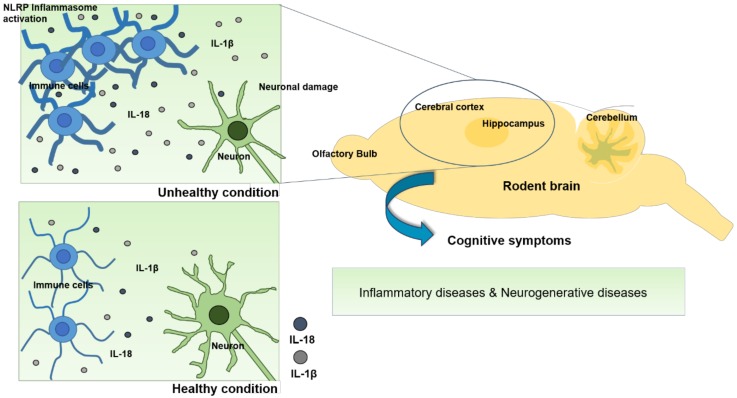
Imbalanced or dysregulated inflammasomes are linked to inflammatory, and neurodegenerative diseases. In these diseases, activation of inflammasome complex and pro-inflammatory cytokines IL-18 and IL-1β in immune cells are involved in neuronal damage and degeneration in the brain, in particular, hippocampus and cerebral cortex. These outcomes may be related to cognitive symptoms. Therefore, understanding the inflammasome regulatory mechanisms may be required for the prevention and treatment of cognitive symptoms.

In this review, we summarized the accumulated evidence on the relationships between the inflammasome and cognitive symptoms in various disease conditions. Understanding the regulatory mechanisms of the inflammasome is necessary to account for cognitive disorders in inflammasome-driven diseases (Figure 3). A growing number of studies have revealed that the manipulation of inflammasome components by genetic or pharmacological tools can restore cognitive dysfunction. MCC950, VX-765, Ac-YVAD-CMK, or z-YVAD-fmk, which can downregulate inflammasome components, exert beneficial effects on both cognition and pathophysiological features in human diseases, such as inflammatory and neurodegenerative diseases. These observations suggest that the development of drugs targeting the inflammasome can prevent a critical step in the progression toward cognitive symptoms. In conclusion, biological evidence offers an important perspective on the relationships between inflammasome and cognitive symptoms in the pathophysiology of several diseases and suggests a significant potential relevance of the inflammasome in preventing and treating cognitive symptoms.

## Abbreviations

6-OHDA	6-hydroxydopamine
Aβ	β-amyloid
Ac-YVAD-CMK	Ac-Tyr-Val-Ala-Asp-chloromethylketone
AD	Alzheimer’s disease
AIM2	absent in melanoma 2
ALR	AIM2-like receptor
APOE	apolipoprotein E
APP	amyloid precursor protein
ASC	apoptosis-associated speck-like protein containing a carboxy-terminal CARD
BACE1	beta-site APP cleaving enzyme
BBB	blood-brain barrier
CARD	caspase activation and recruitment domain
CLP	cecal ligation and puncture
CLR	C-type lectin receptor
CNS	central nervous system
CT	computerized tomography
DAMP	danger-associated molecular pattern
EAE	experimental autoimmune encephalomyelitis
GABA	γ-aminobutyric acid
GSDMD	gasdermin D
GM-CSF	granulocyte-macrophage colony-stimulating factor
IFN	interferon
IL	interleukin
IL-1R	IL-1 receptor
IL-1RA	IL-1R antagonist
LPS	lipopolysaccharide
LRR	leucine-rich repeat
LTP	long-term potentiation
MAP	microtubule-associated protein
MCP-1	monocyte chemoattractant protein-1
MPTP	1-methyl-4-phenyl-1,2,3,6-tetrahydropyridine
MRI	magnetic resonance imaging
MS	multiple sclerosis
NACHT, NBD	nucleotide-binding domain
NADPH	nicotinamide adenine dinucleotide phosphate
NF-κB	nuclear factor kappa-light-chain-enhancer of activated B cells
NLR	NOD-like receptors
NOD	nucleotide-binding oligomerization domain
PAMP	pathogen-associated molecular pattern
PD	Parkinson’s disease
PND	perioperative neurocognitive disorder
POCD	postoperative cognitive dysfunction
PYD	pyrin domain
PRR	pattern recognition receptor
PSEN	presenilin
RIG	retinoic acid-induced gene
RLR	RIG-I-like receptor
ROS	reactive oxygen species
SAE	sepsis-associated encephalopathy
Sirt1	sirtuin 1
Th	T helper
TLR	Toll-like receptor
TNFR	tumor necrosis factor receptor
VACV	vaccinia virus
